# Potential infection foci in the oral cavity and their impact on the formation of central nervous system abscesses: A literature review

**DOI:** 10.1097/MD.0000000000035898

**Published:** 2023-11-17

**Authors:** Kamil Abed, Marcin Paciorek, Dominik Bursa

**Affiliations:** a Department of Cranio-Maxillofacial and Oral Surgery and Implantology, Infant Jesus Clinical Hospital-University Clinical Center of the Medical University of Warsaw, Warsaw, Poland; b Department of Adult Infectious Diseases, Medical University of Warsaw, Warsaw, Poland.

**Keywords:** brain abscess, neurosurgery, odontogenic brain abscess, odontogenic foci

## Abstract

Despite advancements in preventive, diagnostic, and therapeutic activities in medicine, inflammatory processes of the central nervous system remain a significant problem, posing a serious threat to life and health. Purulent central nervous system infections are unique, including abscesses of the brain and spine, which are severe infections occurring in 0.4% to 0.9% of 1000 patients worldwide. Central nervous system abscesses have varying etiology. For example, organized, encapsulated abscesses of the brain are a unique group of inflammatory processes in the central nervous system caused by inflammation around the teeth in 3% to 10% of cases. Sometimes, the condition of patients with brain abscesses is severe and life-threatening. Therefore, detecting and eliminating all causes early, including those potentially resulting from odontogenic infections, is important; accurate and early diagnosis enables appropriate treatment. This paper presents a review of the information available in the literature on brain abscesses and their relationship with odontogenic foci of infection in the oral cavity.

## 1. Introduction

The oral cavity is a unique environment with the second largest and most diversified microflora after the intestines, containing > 700 species of bacteria.^[1]^ Its contact with the air, food, and water makes it a dynamic, diverse, and unique environment for microorganisms. Microorganisms live in various parts of the oral cavity, such as the teeth, periodontal pockets, tonsils, mucous membranes, soft and hard palates, and tongue, which make it an ecologically complex system known as the oral microbiome.

Bacteria are dominant components of the oral microbiome. They are mainly gram-positive granulomas from the *Streptococcus* genus (*S. mutans, S. salivarius, S. milleri,* and *S. oralis*) and bacteria from the *Abiotrophia* and *Staphylococcus* genera.^[2]^ More than 1200 different types of microorganisms have been identified in the oral cavity, of which 350 and 150 bacterial strains have been isolated in marginal and periapical periodontitis cases, respectively.^[3]^ The oral microbiome is also composed of other forms of microorganisms, including viruses (Type 1 and 2 Herpes simplex, Cytomegalovirus, Human papillomavirus, and Epstien-Barr virus), protozoa (*Entamoeba gingivalis* and *Trichomonas tenax*) and fungi from the *Candida* genus (*C. albicans*).^[2]^ These domains of the oral microbiome are important for the proper functioning of the body, including maintaining its immune status.

Factors such as poor hygiene, smoking, and overuse of antibiotics, toothpaste, and mouth rinses can disturb the natural biological balance of the oral microflora and consequently lead to tooth decay, gingivitis, periodontitis, candidiasis, dental pulp diseases, and oral cancer.^[4]^ Dental caries and periodontal disease are common oral diseases caused by the dominance of acid-forming and acid-loving bacteria, such as *Streptococcus mutans*. If left untreated, they are a potential source of infection that can lead to serious infectious complications in the head and neck. The primary foci of infection may be in various body locations, such as the appendages, urinary tract, or bile ducts. However, up to 90% of infections often spread from the head.^[5]^ The middle ear, paranasal sinuses, and in particular, inflammatory reactions within the oral cavity, including the tonsils, teeth, and periodontium, account for 80% of all foci developing both iatrogenically and after proper treatment, such as procedures like tooth extractions and endodontic treatment.^[3,5,6]^

Odontogenic infections are unique, often resulting from complications due to untreated caries or periodontal diseases and are a common cause of dental visits. They may be caused by untreated dental caries, inflammation, pulp necrosis, failure of endodontic treatment, inflammation of the marginal and apical periodontium, impacted teeth or difficult eruption, root apex resection complications, purulent cysts, bone inflammation, and leftover roots fragments after extraction.^[6]^ These infections are usually locally limited but spread rapidly under certain conditions, occupying subsequent anatomical spaces within the head and neck.^[7,8]^ Numerous severe complications accompanying odontogenic infections have been documented in the literature, including cellulitis, osteitis, sinusitis, cavernous sinus thrombosis, mediastinitis, necrotizing fasciitis, empyema, sepsis, meningitis, and abscesses within the central nervous system (CNS).^[7,9]^

The spread of infection depends on the balance between the patient’s general condition and the virulence of the microbial organism present. The virulence of microorganisms and the local and general conditions of the patient determine host immunity. The patient’s general condition in diseases such as HIV/AIDS, uncontrolled diabetes, immunodeficiency, alcoholism, or cachexia favors the spread of the infection.^[10]^

Oral bacteria can spread to the brain and cause life-threatening infections. Hence, treating patients for organized and encapsulated CNS abscesses, including brain abscesses, presents a challenge.

The aim of this article is to present a comprehensive literature review on the etiology, pathophysiology, clinical presentation, diagnosis, and treatment of brain abscesses in the aspect of the presence of potential foci of infection in the oral cavity. The article discusses the challenges associated with the diagnosis and treatment of odontogenic brain abscesses. Additionally, the study highlights the significant role of dentists and maxillofacial surgeons in diagnosing oral cavity inflammation and preventing brain abscesses by maintaining proper oral hygiene and the elimination of infection sources, such as sinusitis.

## 2. Methods

Data from 2000 to 2023 was collected from the PubMed medical database for this review. Manuscripts published before 2000 were omitted; only original articles, case reports, and reviews published in the last 23 years were focused on. The search terms were: “brain abscess odontogenic origin” and “brain abscess dental infection.” There were 29 cases of “brain abscess of odontogenic origin” and 28 cases of brain abscess due to dental infection.” In total, 45 articles were included in the final analysis.

## 3. Results

### 3.1. Epidemiology

CNS inflammation is a significant health problem that seriously threatens life and health. Purulent CNS infections are unique, including severe infections such as brain abscesses. They are reported to have an incidence rate of 0.4% to 0.9% per 1000 people worldwide.^[11]^ Shibata et al reported the incidence of brain abscesses as 0.4 to 0.9 cases per 100,000 persons.^[12]^ Brain abscesses can occur at any age, but most cases occur between the third and fifth decades of life,^[13]^ and more often affecting males < 60 years old.^[14]^ Additionally, 25% of all brain abscesses occur in children between 4 and 7 years old, often in multifocal locations (about 30% of cases).^[15]^

With medical advancement, modern neuroimaging techniques such as computed tomography (CT) and magnetic resonance imaging (MRI), improved surgical techniques, and more effective antibiotic therapy are available. However, these infections are still associated with a high mortality rate of 10%^[11,14,16–18]^ up to 24%^[14,19,20]^ and serious complications that require prompt and effective treatment.^[21]^ Boatright et al^[22]^ estimated that the mortality rate among patients diagnosed with a brain abscess is between 17% and 37%. Cruyssen et al^[23]^ mortality due to odontogenic brain abscesses ranges from 0% to 24%.

See Supplemental Digital Content, http://links.lww.com/MD/K583. Text which describes the aetiology of the brain abscesses

### 3.2. Pathogenesis

Although the frequency of odontogenic CNS infections is not precisely known, inflammatory processes within the oral cavity, including dental caries, periodontal abscesses, gingivitis, and periodontitis, must be considered potential starting points for this location due to their high prevalence. Clifton and Kalamchi defined organized, encapsulated brain abscesses as a unique group of inflammatory processes in the CNS; in 3% to 10% of cases, the cause of this is considered inflammation of the periodontium.^[24,25]^ Jung et al showed that odontogenic infections are a blood-borne cause of brain abscesses, accounting for < 2% of cases.^[14,26]^ Recent dental treatments, such as tooth extraction and caries treatment, as well as poor oral hygiene and diseases, such as diabetes, are known risk factors for developing brain abscesses due to transient bacteremia associated with a decrease in immunity.^[14]^

Clancy et al reported a rare parietal lobe abscess caused by *Actinomyces meyeri* 7 days after tooth 37 (mandibular left second molar) extraction in a generally healthy 55-year-old female.^[27]^ Another paper presented a case of a brain abscess in the frontoparietal region caused by *Streptococcus intermedius* in a 7-year-old, previously healthy child 3 weeks after dental treatment of the left maxillary deciduous molar.^[28]^ This proves that inflammatory changes in the oral cavity, such as caries in extracted teeth, increase the risk of bacteremia and metastatic changes in the form of brain abscesses. Cases of brain abscess in patients who had not previously undergone dental procedures have also been reported. Park et al^[29]^ reported a case of a temporal lobe brain abscess in a 53-year-old female patient with chronic periodontitis in the region of the last molar in the right jaw without symptom exacerbation; the tooth was qualified for extraction. This case suggests that a dental procedure is unnecessary for spreading the inflammatory process from the oral cavity to the CNS.

Recently, inflammatory diseases of the periodontium have been considered important as odontogenic factors in the formation of brain abscesses. Patients with periodontal disease are at risk of developing bacteremia while performing routine hygienic activities, such as brushing teeth and removing tartar deposits.^[30]^

In a meta-analysis, Tomás et al demonstrated a significant increase in plaque accumulation and gingival inflammation scores following tooth brushing, flossing, and chewing, leading to an elevated prevalence of bacteremia.^[31,32]^

Frequent bacteremia and systemic release of proinflammatory cytokines from periodontal pockets can lead to the release of leukocyte elastase and acute phase proteins.^[32]^

However, there is no substantial evidence to suggest that everyday oral activities increase the likelihood of systemic bacteremia.^[31]^

Bacteremia associated with inflammatory periodontal diseases may also be associated with the formation of brain abscesses and many other systemic diseases, such as infective endocarditis, meningitis, subdural empyema, and atherosclerosis.^[30]^

More recent evidence suggests that *cumulative* bacteremia resulting from repetitive, everyday oral activities such as chewing, toothbrushing, and dental flossing may be a more likely cause than a single dental procedure, particularly if high levels of virulent pathogens are present (i.e., in an unhealthy mouth).^[31]^

Other potential odontogenic causes that may influence the formation of brain abscesses are purulent cysts and inflammation of the periapical periodontium.^[14]^

Inflammatory processes from the oral cavity spread to the cranial cavity via four possible routes: systemic bacteremia (hematogenous); direct venous drainage through the facial veins and pterygoid plexus to the cavernous sinus; inoculation via contiguous extension; and lymphatic drainage.^[3,33]^ Akashi et al^[33]^ reported that bacteremia via the hematogenous route is more important in the spread of inflammatory processes to the inner skull than direct venous drainage. This hypothesis is supported by the previous finding that the incidence of intracranial infection was equally likely in the presence of an infection focus in either the maxilla or the mandible.^[33]^ Brain abscesses in the posterior part of the cranial cavity suggest that the hematogenous pathway plays a much greater role in their formation than direct venous drainage.^[3]^

The spread of inflammatory processes by continuity applies to odontogenic inflammation, sinusitis, ear, or mastoiditis, ranging from 14% to 58%.^[13]^ Depending on the source of the infection and the path of the spread of the inflammatory process, the location of brain abscesses may vary. The most common locations of brain abscesses are the temporal lobe (42%) and the cerebellum.^[13,19,34]^ This mainly applies to brain abscesses resulting from subacute or chronic otitis media or mastoiditis.^[24,35]^ When the source of infection is the oral cavity or paranasal sinuses, the frontal lobe is the most common location of a brain abscess.^[13,35]^ The increased risk of odontogenic brain abscesses is attributed to the inflammatory processes in the molars, maxilla, and mandible.^[3,22]^ It is possible for the inflammatory processes to spread from the oral cavity to the inner skull through the continuity of orbital soft tissues.

Purulent processes within the maxillary teeth may spread through the maxillary sinus or buccal soft tissues to the inside of the orbit, causing inflammation within the tissues. Inflammatory processes may further penetrate the skull from the orbit through the superior orbital fissure, causing serious complications. This is most often the method of spreading through tissue infiltration or local venous plexuses.^[36]^

Brain abscesses are usually located on the same side as the infection’s intraoral focus when spreading through the continuous inflammatory processes from the oral cavity. Moazzam et al reported unilateral localization of brain abscesses and foci of infection in the oral cavity in 70% of the cases.^[3]^ Akashi et al^[33]^ also noted that odontogenic foci of infection in the oral cavity on one side might also cause brain abscesses on the opposite side. This confirms the blood-borne path of spreading inflammatory processes from the oral cavity to the inner skull.

See Supplemental Digital Content, http://links.lww.com/MD/K584. Text which describes the microbiology of the brain abscesses.

### 3.3. Clinical presentation

The clinical presentation of intracranial abscesses is variable and nonspecific. The characteristics of symptoms depend on the origin of infection and virulence of the pathogen, site, size, number of lesions, affected brain structures, and the patient’s immune status.^[37]^ The clinical course of odontogenic brain abscesses does not usually differ significantly from brain abscesses of other origins.^[36]^ Frequently, patients present with symptoms such as increased intracranial pressure (ICP), i.e., headache (49–93%), impaired consciousness (33–70%), nausea and vomiting (26–71%), focal neurological symptoms (29–71%), and epileptic seizures (2–49%).^[13]^ Another common symptom is fever (14–88%).^[13]^ Nathoo et al^[37]^ reported that headache, fever, and neck stiffness were the most common clinical symptoms. Additionally, Patel et al^[13]^ reported that the classic triad of symptoms, including fever, headache, and focal neurological deficits, suggested the occurrence of an intracranial abscess in the minority (2–34%).

Patients with brain abscesses usually report one or more symptoms, such as headache, fever, and altered consciousness, or focal neurological symptoms, including seizures, balance disorders, dysphagia, or focal sensorimotor deficits. Frontal lobe abscesses often cause nonspecific and subtle clinical symptoms. Headache, fever, and nausea occur in the early stages, and symptoms of increased ICP, such as impaired consciousness, appear later.^[35]^ Lesions in the occipital lobe may rupture into the ventricle, causing ventricular or lining inflammation or septic thrombophlebitis of the transverse sinus, resulting in venous hypertension, edema, seizures, and increased ICP.^[35]^

It should be considered that frequent oral antibiotic or analgesic prescriptions may temporarily relieve symptoms but prolong the disease course. In addition, immunological disorders may mask symptoms,^[38]^ and the symptom duration varies from 1 day to 8 weeks.^[35]^ The average time from symptom onset to reporting to a doctor was 7 to 25 days.^[13]^ Infections that spread continuously through the orbit or soft tissues of the face have a characteristic course in which there are strongly expressed symptoms associated with phlegmon in the tissues of the orbit and face or inflammation of the paranasal sinuses.

### 3.4. Diagnosis

#### 3.4.1. Imaging.

MRI is the preferred imaging method because it is more sensitive and specific than CT.^[13,38]^ MRI enables the early detection of encephalitis, better visualization of dissemination to the ventricles and subarachnoid space, and the presence of satellite lesions.^[38]^ When an MRI examination is impossible, two-stage CT with or without a contrast agent is the second choice in case of a suspected brain abscess. CT facilitates early detection, accurate localization, and accurate characterization and determination of the number, size, and staging of abscesses.^[39]^ It also detects hydrocephalus, elevated ICP, edema, and associated infections such as subdural empyema and ventriculitis. Thus, it helps in treatment planning, assessment of treatment adequacy, and sequential follow-up.

The first radiological sign of a brain abscess can be observed on CT 2 to 3 weeks after infection.^[24]^ The typical finding of a brain abscess on CT or MRI is a hypodense lesion with a contrast-enhanced ring around the abscess.^[39]^ Other imaging tools used to diagnose brain abscesses include diffusion-weighted imaging and proton magnetic resonance spectroscopy, which allow the differentiation of brain abscesses from cystic tumors.^[13]^

If an odontogenic cause of brain abscess is suspected, a pantomogram or cone-beam CT should be performed to exclude potential foci of infection in the oral cavity.

#### 3.4.2. Laboratory data.

Laboratory tests have limited usefulness in the diagnosis of brain abscesses.^[13]^ Leukocytosis, elevation erythrocyte sedimentation rate, and elevated C-reactive protein levels are common abnormalities in laboratory tests. However, the absence of these abnormalities does not exclude the diagnosis.^[13]^ Some patients have leukopenia in laboratory tests. Blood cultures should be performed as early as possible in all patients with suspected brain abscesses, although only 14–50% of blood cultures aid identification of the pathogen.^[13]^

Cerebrospinal fluid (CSF) analysis reveals pleocytosis, elevated protein levels, and decreased glucose levels. However, the CSF is unchanged in most patients, and CSF cultures are rarely positive (0–43%).^[13]^ It should be emphasized that brain abscesses are the only CNS infection for which lumbar puncture is never recommended and may be contraindicated. This is because lumbar puncture may lead to deterioration of the neurological condition and, in extreme cases, may cause death due to brain herniation.^[35,38]^

See Supplemental Digital Content, http://links.lww.com/MD/K585. Text which describes the differential diagnosis of brain abscesses.

### 3.5. Treatment

In most cases, the management of brain abscesses combines surgery (drainage, excision, or stereotactic aspiration) and pharmacological treatment (oral, intravenous, or intrathecal antibiotics).^[40]^

All patients diagnosed with brain abscesses should receive empirical antibiotic therapy, including treatment for aerobic and anaerobic pathogens.

To select the most appropriate drug, the pharmacokinetic and pharmacodynamic characteristics of each compound, previous treatment, administration routes, patient’s predisposing conditions, potential pathophysiological mechanism underlying the abscess, and the microorganism involved must be considered.^[40]^

Initial therapy should be initiated with broad-spectrum antibiotics that can cross the blood-brain and blood-CSF barriers at appropriate concentrations. Most authors recommend the routine use of third- and fourth-generation cephalosporins. For example, ceftriaxone 2 g twice daily, in combination with metronidazole 500 mg 4 times daily, and vancomycin 1 g 3 times daily if there is a history of penetrating injury or recent neurosurgery.^[22,35]^ Immunocompetent patients with immunodeficiencies, such as HIV/AIDS, are at risk of brain abscesses caused by *Nocardia asteroides* or *Toxoplasma gondii*. In cases of toxoplasmosis, patients were treated with voriconazole, pyrimethamines, such as daraprim and sulfadiazine, or clindamycin.^[13,40]^

The success rates for antibiotic therapy are higher when the treatment is initiated during early stage cerebritis, if the lesion measures < 1.5 cm diameter, in patients with progression times < 2 weeks, and in patients displaying symptom improvement within a week of treatment.^[40]^

Patients receiving immunosuppressant or immunomodulatory drugs should receive empirical treatment with third generation cephalosporins (ceftriaxone or cefotaxime); further, fourth generation cephalosporins (cefepime) should be administered if Pseudomonas infection is suspected, and combined with metronidazole, vancomycin, or trimethoprim-sulfamethoxazole (the latter in cases of suspected Nocardia spp. infection).^[40]^

The best solution is to implement targeted therapy based on the surgically collected samples’ microbial culture and sensitivity results. Arlotti et al^[41]^ recommended collecting the culture sample no later than 3 days after commencing antibiotic therapy or before its initiation. This increases the chances of isolating the pathogen from the collected material. If the culture of the collected material is negative, broad-spectrum antibiotics are administered. Intravenous antibiotic therapy is continued for 6 to 8 weeks, and oral antibiotic therapy should be continued after completing intravenous therapy for another 2 to 3 months.^[13]^ With appropriate antibiotic therapy, the abscess should decrease within 1 to 4 weeks; however, it may take up to 16 weeks for the radiological changes to disappear.^[22]^

Depending on the case, the treatment method choice should always be considered individually.

It remains debatable as to when only surgical treatment should be used and when it should not.

In cases of brain abscesses smaller than 1 cm, Greggory D Boatright II et al recommend a conservative treatment i.e., antibiotic therapy without the need for surgical treatment.^[22]^

Hernando Alvis Miranda et al referred to the study by Arlotia et al in their investigation of brain abscesses, and stated that they believed that the individuals with a small brain abscess of less than 2.5 cm are best candidates for conservative treatment, accompanied with well-known etiology and good clinical condition.^[35]^

The eligibility criteria for non-surgical treatment according to M.A. Ruiz-Barrera et al include the presence of multiple brain abscesses with a diameter of < 1.5 cm, presence of a single lesion measuring < 1.5 cm diameter, lesion location in eloquent areas of the brain (ex.left temporal and frontal lobes for speech and language), and presence of concomitant infections (e.g., meningitis, ependymitis).^[40]^

The availability of modern neuroimaging techniques and use of improved, minimally invasive surgical techniques allow to reduce the size of the abscess and determine its microbiological characteristics.

Drainage can be performed using the open method (craniotomy) or minimally invasive techniques. Open surgery (craniotomy) focuses on drainage of purulent material and excision of the capsule, which minimizes the risk of recurrence. Stereotactic aspiration is increasingly used for abscess drainage. However, the choice between open surgery (craniotomy) and minimally invasive techniques should be made on an individual basis.

For single abscesses > 1 cm, the treatment of choice is surgical treatment using minimally invasive techniques, such as biopsy and drainage using stereotactic techniques or biopsy and drainage under CT control.^[22,40,42]^ The procedure is relatively safe and allows obtaining samples for the examination, while relapses are quite frequent, affecting up to 24% of patients.^[38]^

The advantage of this method is the possibility of repeating it if the first attempt is unsuccessful.

With stereotactic surgery and neuronavigation, aspiration can be performed regardless of the phase of the abscess, which would enable microbiological and histopathological studies.^[40]^

Consider drainage of the largest lesion in patients with multiple small abscesses. The remaining lesions may be drained if they are surrounded by marked edema, if the patient’s clinical status worsens, and/or when response to antibiotic therapy is insufficient.^[40]^

Intraoperative ultrasound is an alternative technique that may facilitate drainage of the abscess when stereotactic surgery and neuronavigation are not available; however, this technique is not recommended in patients with small, deep-seated lesions. Catheter placement in the central region of the abscess for continuous drainage of purulent material and direct antibiotic administration is aimed at reducing the need for surgical reintervention; however, this approach is no longer recommended for routine use. Reintervention may be necessary due to factors such as inadequate aspiration, absence of a drainage catheter in large abscesses, history of immunosuppression, or inadequate antibiotic therapy, among others.

Yamamoto et al^[40]^ recommend performing a CT study at least once a week for imaging follow-up, depending on the patient’s clinical status.

Treatment of brain abscesses by classic craniotomy is recommended in cases of large abscesses located in the immediate vicinity of the ventricular system, with a risk of puncturing it, multiple abscesses, failed aspiration, post-traumatic abscesses containing bone fragments or foreign bodies, multi-chamber abscesses, gas-containing abscesses, and abscesses with coexisting fistulas (e.g., in association with the congenital cutaneous sinus).^[38,42]^

The classic craniotomy treatment of abscesses is indicated in the following cases by M.A. Ruiz-Barrera et al:^[40]^ lesions measuring > 2.5 cm, midline shift > 5 mm, proximity to the ventricular system, and brain herniation. Complete surgical resection of the abscess may be indicated in the following cases: abscesses located in a superficial, non-eloquent area; suspected fungal, Mycobacterium tuberculosis, Actinomyces spp., or Nocardia spp. infection; abscesses resulting from a congenital or acquired fistulous path; multiloculated abscesses; abscesses caused by parameningeal septic foci; and failure of previous treatments.

Optimal management typically requires a combination of antibiotic therapy and surgery. However, in some cases, only conservative treatments can be administered. This applies to internal medicine patients in whom the risk associated with surgery is high, in stable patients with mild clinical symptoms and patients in extremely severe conditions, and in cases of brain abscesses < 1 cm in patients with good clinical condition (Glasgow Coma Scale score > 12).^[22,35,38,42]^

Patients treated for brain abscesses with suspected odontogenic etiology require thorough dental and radiological examinations of the oral cavity. It should be emphasized that the decision to radically or more conservatively treat the focus of infection in the oral cavity by dental treatment depends on the patient’s general health, severity, and type of the disease in the oral cavity, proper oral hygiene, and patient’s understanding of the legitimacy of dental treatment. In particular, dental treatment should be less radical and more conservative to protect the teeth when a patient’s health condition allows it. Many dental treatments can be offered in such situations, including periodontal surgery, root canal treatment, endodontic surgery, and conservative dentistry.^[19]^

In the case of a lack of patient cooperation during dental treatment, noncompliance with oral hygiene, and follow-up visits, the implementation of radical dental procedures, that is, multiple tooth extractions, should be considered.

## 4. Discussion

Untreated inflammation in the oral cavity can lead to serious health-threatening complications, including cellulitis, thrombophlebitis, mediastinitis, necrotizing fasciitis, empyema, phlegmon of the face and neck, sepsis, meningitis, and CNS abscesses. In patients with brain abscesses, the foci of infection in the oral cavity should always be considered a potential source of infection. Ewald et al proposed three criteria for the diagnosis of brain abscesses of odontogenic etiology: no alternative source of bacteremia must be found; microbiological examination of the cultures reveals pathogens typically found in the oral microflora; and clinical or radiographic signs of active dental or periodontal disease must be present.^[17]^

Moazzam et al additionally indicated the potential impact of dental procedures performed, even in the absence of pathology in the oral cavity, which may cause CNS infections, including brain abscesses, especially if performed within 1 to 4 weeks before the onset of the disease.^[3]^ The most common odontogenic factors contributing to the formation of brain abscesses are caries complicated by periapical inflammation and periodontitis. Dental procedures, particularly tooth extraction, including those of wisdom teeth, may also precede and contribute to the development of brain abscesses. There was no predilection for maxillary or mandibular teeth.

Brain abscesses due to dental diseases may predispose some patients to heart defects with a right-to-left shunt, as seen in patent foramen ovale, thoracic venous anomalies, Ebstein anomaly, and venous atrial septal defects.^[22]^ Dental procedures, such as tooth scaling, may increase the risk of brain abscesses in these patients. According to the literature, the source of infection cannot be determined in 10–60% of patients diagnosed with a CNS abscess^[42,43]^; Ewald et al referred to these types of abscesses as cryptogenic or idiopathic.^[17,24,33]^ The location of the brain abscess does not necessarily correlate with the site of infection in the oral cavity, suggesting that the bacteria most likely enter the skull by hematogenous spread rather than by venous drainage.^[3]^ The hematogenous route is considered the most important mechanism for the spread of inflammatory processes from the oral cavity to the inside of the skull with the formation of brain abscesses.^[24,33]^ The average time from the dental procedure to the onset of neurological symptoms is about 2 to 3 weeks.

The initial complaints may be mild and usually include headaches, bad moods, and apathy. Most patients with odontogenic brain abscesses present with neurological symptoms such as seizures, hemiparesis, progressive leg weakness, and speech problems.^[19,44]^ Dental caries and periodontal disease appear to be rare causes of intracerebral or intravertebral infections in healthy individuals; however, the oral cavity should always be considered a potential source of infection in evaluating and treating idiopathic brain abscesses. Patients with immunodeficiency secondary to HIV infection, alcohol abuse, diabetes, chemotherapy, or malignancy are at a higher risk of developing brain abscesses.^[24]^ Several cases of brain abscesses have been reported in previously healthy, immunocompetent patients with poor oral hygiene or after dental treatment.^[24]^

Determining the cause of odontogenic brain abscesses requires the maxillofacial surgeon and dentist to know the basic mechanisms of spreading inflammatory processes from the oral cavity to the inside of the skull.^[33]^ Very often, it is not possible to establish direct evidence that the brain abscess originated from an odontogenic focus because, in many cases, it is not possible to find an active focus of infection in the oral cavity. Patients with a brain abscess present with neurological symptoms long after dental treatment of suspected foci of infection in the oral cavity. Diagnosing a brain abscess of odontogenic etiology is often based on excluding or eliminating other sources of infection, such as sinusitis and otitis media. The most reliable way to confirm the dental etiology of a brain abscess is to compare the culture results of bacterial samples obtained from the brain abscess and the extracranial sites of infection using DNA analysis.^[18,24]^

After establishing the diagnosis, the treatment consisted of three stages: pharmacotherapy, abscess drainage, and treatment of the primary focus. Pharmacotherapy is primarily based on intravenous administration of a combination of broad-spectrum antibiotics. Targeted antibiotic therapies can be administered after identifying the pathogen and obtaining an antibiogram. Steroids are generally not indicated except in patients with life-threatening cerebral edema and mass effects, neurological deterioration, and meningitis symptoms.^[38,39]^ Epileptic seizures occur in 25% to 50% of patients with brain abscesses.^[42]^

Anticonvulsant treatment for 5 years was indicated for all patients with brain abscesses. Antiepileptic drugs may be discontinued if the patient is seizure-free for ≥ 2 years after surgery and electroencephalography does not show epileptic activity.^[39]^ Optimal management typically requires a combination of antibiotic therapy and surgery. Surgical treatment includes minimally invasive techniques, such as biopsy and drainage using stereotactic techniques or CT-guided biopsy and drainage.^[22,42]^ Treatment of brain abscesses by classical craniotomy is recommended in cases of large abscesses located in the immediate vicinity of the ventricular system, multiple abscesses, failed aspiration, post-traumatic abscesses containing bone fragments or foreign bodies, multi-chamber abscesses, gas-containing abscesses, and abscesses with coexisting fistulas (e.g., congenital cutaneous sinus).^[38,42]^

However, depending on the case, the treatment method choice should always be considered individually.

## 5. Conclusions

CNS inflammation is a significant health problem with adverse health effects. Purulent CNS infections are unique, including severe infections such as brain abscesses. The etiology of primary CNS abscesses is diverse. Inflammatory processes within the oral cavity, including dental caries, periodontal abscesses, gingivitis, and periodontitis should be considered due to their high incidence, although they are rarely the potential starting points for intracerebral infections, including brain abscesses in healthy individuals. The oral cavity should always be considered as a potential source of infection in the evaluation and treatment of idiopathic brain abscesses. In many cases, it is not possible to find an active focus of infection in the oral cavity; thus, no direct evidence of the odontogenic etiology of the brain abscess is determined. Even if the brain abscess is of a dental origin, the preceding dental treatment may be distant, making it difficult to prove a causal relationship. Diagnosing a brain abscess of odontogenic etiology is often based on excluding or eliminating other sources of infection, such as sinusitis and otitis media.

Prevention of brain abscesses includes maintaining proper oral hygiene, eliminating foci of infection in the oral cavity, and treating sinusitis. Regular tooth cleaning, dental office radiography, and awareness of oral infections can prevent the brain abscesses from spreading.

Dentists play an integral role in diagnosing oral inflammation that can spread to the brain. A thorough dental examination of the oral cavity and diagnostics using radiological images in dental clinics allows early detection of potential foci of infection in the oral cavity, thereby enabling early prevention and treatment of brain abscesses.

## Author contributions

**Conceptualization:** Kamil Abed.

**Formal analysis:** Marcin Paciorek, Dominik Bursa.

## Supplementary Material






