# Supplementary material for: Potential infection foci in the oral cavity and their impact on the formation of central nervous system abscesses: A literature review
Source: Medicine (Baltimore). 2023 Nov 17;102(46):e35898. doi: 10.1097/MD.0000000000035898 (PMC10659677; doi:10.1097/MD.0000000000035898)
Supplement: Supplementary file 1 [file medi-102-e35898-s001.docx]

Supplemental content_which describes the aetiology of the brain abscesses_1

**Aetiology**

CNS abscesses most often result from craniocerebral trauma or neurosurgery. These are also known as secondary abscesses, which have been previously reported to occur in 8–19% of cases,^24,25^ and 10% of cases in other reports.^26^

The aetiology of primary CNS abscesses is diverse. However, continuous inflammatory processes play an important role, spreading from the paranasal sinuses, the middle ear, and odontogenic foci of infection in the oral cavity. They account for 25–50% of cases and are usually solitary brain abscesses.^25,26^

Inflammatory processes from other locations, such as lung abscesses or empyema, bacterial endocarditis, skin infections, and the abdominal cavity (including the pelvis), are spread by blood-borne dissemination.^24,25,27^ This spread of inflammatory processes into the cranial cavity is estimated to have a 25% prevalence.^26^ Many haematogenous brain abscesses are multifocal and centred in the middle cerebral artery distribution, especially in patients with cyanotic heart disease.^25^

According to the existing literature, the source of infection cannot be determined in 10–60% of patients diagnosed with a CNS abscess^28,29^; this type of brain abscess is referred to as “cryptogenic” or “idiopathic” abscesses.^26,30^
