# Supplementary material for: Potential infection foci in the oral cavity and their impact on the formation of central nervous system abscesses: A literature review
Source: Medicine (Baltimore). 2023 Nov 17;102(46):e35898. doi: 10.1097/MD.0000000000035898 (PMC10659677; doi:10.1097/MD.0000000000035898)
Supplement: Supplementary file 2 [file medi-102-e35898-s002.docx]

Supplemental content_which describes the microbiology of the brain abscesses_2

**Microbiology**

The microbiological flora present in brain abscesses varies. Brain abscesses of odontogenic aetiology are caused by many microorganisms, predominantly bacteria from the *Streptococcus* genus, particularly gram-positive *Streptococcus viridans* (*S. anginosus*, *S. constellatus*, and *S. intermedius*), *Actinomyces*, and *Staphylococcus aureus*.^30,38^ It is estimated that oral flora have been found in approximately 3–10% of all cases of brain abscesses, with the rest being non-oral bacteria, *Mycobacterium tuberculosis*, fungi, and parasites.^26^

Many authors have pointed to an anaerobic aetiology of brain abscesses, mainly from organisms such as *Peptostreptococcus*, *Bacteroides fragilis*, *Prevotella*, *Enterobacteriaceae*, *Fusobacterium* and gram-negative *Aggregatibacter actinomycetemcomitans* and *Eikenella corrodens*.^30^ Akashi et al. isolated *S. constellatus*, *Fusobacterium nucleatum*, *Parvimonas micra*, *Porphyromonas gingivalis*, and *Lactobacillus catenaformis* from frontal lobe abscesses in three cases.^30^ In all three cases, the source of intracranial infection was teeth with advanced periodontal disease.

Haggerty and Tender reported a case of *Actinomyces* brain abscess following endodontic treatment of the anterior maxillary teeth.^39^ Clancy et al. presented a case of *Actinomyces* brain abscess that occurred 7 days after extraction of the lower left molar.^33^ It should be emphasised that despite the culture taken from the abscess, determining the aetiological factors is not always possible. Culture results are negative in 20–40% of all brain abscesses; this is related to the patient’s previous antibiotic therapy and may explain the lack of bacterial growth.^35^
