# Supplementary material for: Potential infection foci in the oral cavity and their impact on the formation of central nervous system abscesses: A literature review
Source: Medicine (Baltimore). 2023 Nov 17;102(46):e35898. doi: 10.1097/MD.0000000000035898 (PMC10659677; doi:10.1097/MD.0000000000035898)
Supplement: Supplementary file 3 [file medi-102-e35898-s003.docx]

Supplemental content_which describes the differential diagnosis of the brain abscesses_3

**Differential diagnosis**

The differential diagnosis of brain abscesses requires a thorough clinical examination and accurate imaging.

The presence of radiological features of a brain abscess is insufficient for differentiating a common brain abscess from mycotic, nocardial, tuberculous, inflammatory granulomatosis (tuberculoma); neural cysticercosis; toxoplasmosis; glioblastoma; metastatic tumours; resolving hematoma; cerebral infarction; cystic lymphoma; and necrosis.^49^ However, the presence of fever, meningeal irritation (meningism), elevated ESR, multilocularity, reduced ring enhancement in delayed examination, and gas within the lesion supports the diagnosis of an abscess.^49^

During a craniotomy, haemostatic materials such as oxidized cellulose, swabs, gelatine sponge, and microfibrillar collagen are used to control bleeding. Haemostatic materials commonly used for haemostasis can cause a granulomatous reaction, which can lead to foreign body-related inflammatory pseudo-tumors.^48^

Textiloma, gossypiboma, gauzoma, and muslinoma are terms that are used to describe foreign body-related inflammatory pseudo-tumours.^48^

These terms refer to the mass-effect brain lesion caused by leftover absorbable or non-absorbable haemostatic agents used during surgery, together with the associated inflammatory reaction.^48^

Textiloma can show up at any time—initially, they do not cause any clinical symptoms, but they may appear months or even years after surgery. In the case of hyperdense lesions discovered post-surgery, this type of inflammatory pseudotumor associated with foreign bodies left behind should always be considered in the differential diagnosis. They can mimic intracranial tumours, repeated intracranial bleeding in postoperative imaging, postoperative necrosis, and postoperative brain abscesses. ^48^
